# Ensuring continuity of care during the COVID-19 pandemic in Guinea: Process evaluation of a health indigent fund

**DOI:** 10.3389/fpubh.2022.1018060

**Published:** 2022-11-17

**Authors:** Lansana Barry, Mariama Kouyaté, Abdoulaye Sow, Willem Van De Put, John De Maesschalck, Bienvenu Salim Camara, Norohaingo Adrianaivo, Alexandre Delamou

**Affiliations:** ^1^African Center of Excellence for the Prevention and Control of Communicable Diseases, Gamal Abdel Nasser University of Conakry, Conakry, Guinea; ^2^Centre National de Formation et de Recherche en Santé Rurale de Maférinyah, Forécariah, Guinea; ^3^Fraternité Médical Guinée, Conakry, Guinea; ^4^Department of Public Health, Institute of Tropical Medicine, Antwerp, Belgium; ^5^Memisa, Ixelles, Belgium

**Keywords:** continuity of care, COVID-19, pandemic, process evaluation, health indigent fund, Guinea

## Abstract

**Background:**

The emergence of the COVID-19 has disrupted the health and socioeconomic sectors, particularly in resource-poor settings such as Guinea. Like many sub-Saharan countries, Guinea is facing shortcomings related to its fragile health system and is further affected by the passage of the Ebola virus disease. The pandemic has worsened the socio-economic situation of the poorest people, leading to their exclusion from health care. To promote access to care for the most vulnerable populations, a system was set up to provide care for these people who are victims of health marginalization to promote their access to care. This study aimed to analyze access to health services by vulnerable populations during the COVID-19 pandemic in Guinea through the establishment of a health indigent fund (HIF).

**Methods:**

This was a qualitative study to assess the project implementation process. A total of 73 in-depth individual interviews were conducted with beneficiaries, health workers, community health workers and members of the HIF management committee, and a few informal observations and conversions were also conducted in the project intervention areas. The data collected were transcribed and coded using the deductive and inductive approaches with the Nvivo software before applying the thematic analysis.

**Results:**

A total of 1,987 indigents were identified, of which 1,005 were cared for and 64 referred to all 38 intervention health facilities within the framework of the HIF. All participants appreciated the project's social action to promote access to equitable and quality health care for this population excluded from health care services. In addition, the project has generated waves of compassion and solidarity toward these “destitute” people whose main barrier to accessing health care remains extreme poverty. A state of poverty that leads some to sell their assets (food or animal reserves) or to go into debt to ensure access to care for their children, considered the most at risk.

**Conclusion:**

The HIF can be seen as an honest attempt to provide better access to health care for the most vulnerable groups. Some challenges need to be addressed including the current system of acquiring funds before the attempt can be considered scalable.

## Introduction

Access to health care remains one of the major challenges in West Africa. In addition to geographic barriers and quality of care, another important determinant is the economic inability of some populations to afford care. The low standard of living of populations is often cited as a barrier to accessing health care (1, 2).

During the global spread of COVID-19, Africa stands out as the most affected region in the world in terms of income loss for poor households (3). The poverty incidence rate in 2021 is estimated at $1.90 purchasing power parity (PPP) per day, which has increased by 3% due to the COVID-19 pandemic (3). In 2019, 478 million people were living in extreme poverty compared to 490 million people living below the $1.90 PPP/day poverty line in 2021 in Africa, 37 million more than expected without the pandemic (3).

The advent of this pandemic has impacted the health systems of several countries, ranging from the disruption of health services to their under-utilization by the populations, either for fear of being contaminated or for lack of financial means. The impacts of the COVID-19 pandemic extend to the socio-economic and psychological domains, caused by the loss of jobs and the reduction of daily income-generating activities carried out by the populations due to the confinement and the reduction of the number of passengers in public transport (4).

**Figure 1:**
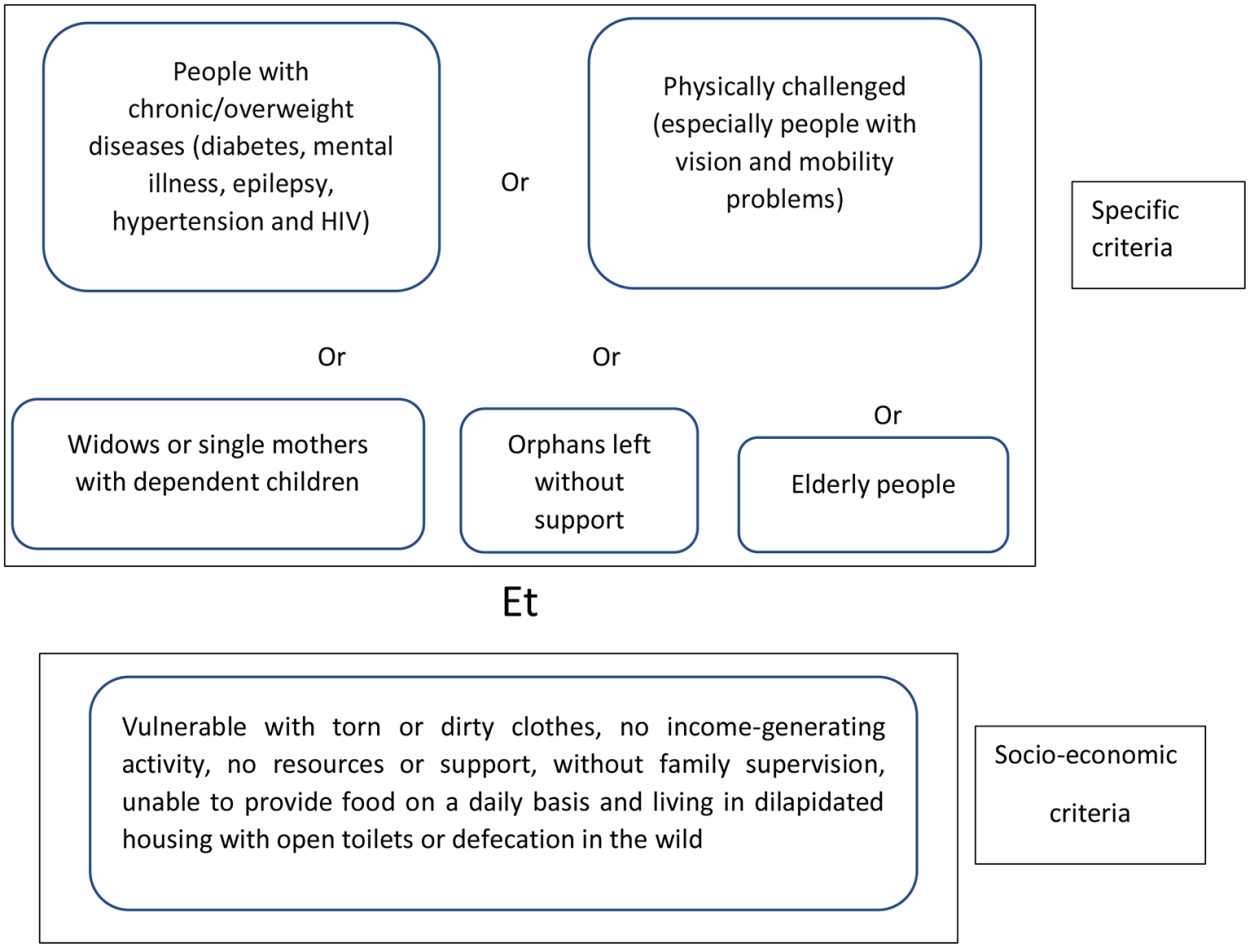
Active form of indigent identification criteria.

Given the impoverishment of the population aggravated by the pandemic and the restrictive measures imposed by the government to control its spread (4), Belgian and Guinean NGOs piloted a financial protection mechanism to promote access to care for vulnerable populations. The pilot was tested in the regions of Conakry, Kindia, Mamou, and Labé. This social protection component is part of a bigger “health system strengthening” project to promote access to care for vulnerable populations. As part of the management of vulnerable persons, a set of mechanisms to facilitate this process has been put in place by the project implementation team. The system, describes the criteria for identification, care and referral of sick people if necessary. For this purpose, potential actors at the heath facility and community levels were identified and organized into a HIF management committee. The role of this committee goes beyond the management of funds. Composed of a local councilor (head of the neighborhood or district), the head of the health facility covering the area, the community representative (a leader such as the Imam) and the CHW, this committee federates their actions in the identification of vulnerable people within each community. Following the vulnerability criteria set up by the project team, the CHW in collaboration with the leader or local councilor proceeds to identify the vulnerable during field visits. The following scheme was adopted by the project as the basis for fieldwork in identifying indigents people (Figure 1).

In addition to this form of active identification, there is another one that is done within the health facility itself with the help of the health workers. There, a selection is made through the client's physical appearance and his reaction to the cost of the medical prescription. If the client is found to be vulnerable, his name is communicated to the CHW, who, then registers the person for health follow-up. The care for vulnerable people began in June 2021, and is known as the “health indigent fund” (HIF). This part of the project was in the context of improving access to health for all—Universal Health Coverage (UHC). Contributing to UHC is part of the Memisa, *Fraternité Médical Guinée* (FMG) and the European Union (EU) interventions. In light of the COVID-19 pandemic and the need for access to health care, the policy of waiving fees to specific segments of the population perceived to be most in need was undertaken.

The objective of this study was to analyze access to and use of health services by vulnerable populations during the COVID-19 pandemic in Guinea through the implementation of a health indigent fund.

## Methods

### Study setting and type of study

The study was conducted in 34 health facilities (11 community health centers in the rural health regions of Kindia, Mamou, and Labé, 17 community health centers in the city of Conakry, and 6 public health centers). All health facilities covered by the project were included for the purpose of this study, and at least one participant was interviewed from each. For this purpose, the maximum variation sampling was used during participants selection. This process took into account the variation of certain characteristics of the participants. For example, for the health workers, we took into account their profession (doctor, nurse, midwife) and their position in the health facility (head of center or deputy); for the CHWs, we took into account their affiliation with the various health centers (private or public); for the beneficiaries and other members of the health indigent fund management committee (leaders and local councilors), we took into account variation in sociodemographic characteristics (age group, gender, proximity to the health facility and area of residence).

We conducted a process evaluation, focusing on the implementation process while attempting to determine the extent to which the project succeeded in following the strategy described in the logic model that was established at the outset through a qualitative study.

### Study participants and recruitment

During this study, we interviewed various stakeholders, including health workers from the private and public sectors, community agents, community leaders or local councilors, and beneficiaries in the various project intervention zones. The recorded interviews were transcribed verbatim. The transcripts were then coded using the deductive and inductive approaches (5). We then conducted a thematic content analysis in which the codes were categorized into subthemes and themes based on similarities and differences. NVivo 12 software was used to analyze the data.

### Data collection technics and tools

For data collection, individual interview guides, a literature review based on project documents, and informal conversations were conducted.

#### In-depth interview

Using the interview guides, in-depth interviews were conducted with participants. The interview guides were composed of different sections: the description of the care system for vulnerable persons with the HIF, compliance with the guide, perceptions of the care process, difficulties and facilitating factors in the functioning of the system, and challenges.

#### Informal conversation

In each collection team, there was at least one sociologist who led informal conversations to further identify challenges and solutions that were being considered during implementation and that some participants would not share during IDIs.

#### Project documents review

A review of the project documents focusing on the tools used for the care of vulnerable (guide for setting up a system to provide access to free or low-cost services for the most vulnerable) was carried out and the information collected was triangulated with the primary data in order to understand and assess the process put in place.

### Analyse des donnees

Interviews were fully transcribed and cross-checked before being imported into NVivo 12 software. The transcripts were then coded using deductive and inductive approaches (5). We then conducted a thematic analysis in which the codes were categorized into subthemes and themes based on similarities and differences.

### Ethical considerations

The research protocol has received approval L-080-CNERS-21 from the National Health Research Ethics Committee of Guinea.

## Results

A total of 73 in-depth interviews were conducted with beneficiaries (6), health workers (7), CHWs (8), community leaders and local councilors (9) and the project team (1) in the various project intervention zones.

The analysis focused on the description of the care system for the vulnerable, the participant's perceptions of the program and the care process, the facilitators and the major challenges encountered during the implementation of HIF activities.

### Health indigent funds

The project recruited staff (one CHW per facility), and collaborated with some community member to facilitate field activities. This helped fill the staffing gap created by the increased workload in the health services included in this project.

In agreement with the heads of the partner health facilities, community leaders/local councilors and CHW in the intervention zones were identified and trained to implement the HIF for vulnerable people. The CHWs and local councilors/community leaders are mandated by the project to combine their efforts in identifying vulnerable people and monitoring their care within their community (10); while the health workers ensure both the identification at the facility level (10), the management of premiums and the actual medical care.

A guide to HIF has been made available to the CHWs, which describes the identification criteria, from taking charge to referring the very sick patients if necessary. For this purpose, potential actors at the facility and community levels were identified and organized into a health indigent fund management committee. The role of this committee goes beyond the management of funds. This committee is composed of a local councilor or community leader, the head of the health facility in the area, the community representative (a leader such as the Imam) and the CHW. It takes concerted action to identify the vulnerable in each community, following the vulnerability criteria established for this purpose.

To do so, the CHW, in collaboration with the community leader or the local councilor, identifies vulnerable people during field visits. In addition, the health agents also identify indigents among patients who arrive for care and who have not been identified in the field. There, sorting is made through the physical appearance of the client and his reaction to the cost of the medical prescription. If they are found to be vulnerable, their name is given to the CHW, who registers them directly into the identification book for follow-up, which is part of a global registry. Therefore, there are no registers that can be traced back to the origin of the identification (active vs. passive). People identified in both processes are registered and should receive transportation and food allowance, full medical care and a lump sum for referral if necessary. Access to and effective use of health services for and by the vulnerable implies the proper functioning of the HIF in place. In this context, it would be useful to understand whether the HIF has functioned according to the project's guidelines.

Those responsible for identifying and caring for the vulnerable reported that transportation, food allowance, medical prescription fees and referrals for the seriously ill are included throughout the care process. Each vulnerable person who falls ill, receives a premium related to these previously mentioned items. Participants were not engaged in follow-up behavior after service but anytime the vulnerable person falls sick, he/she will receive free care once to the health facility.

Some of the expenses covered by the HIF, such as transportation, vary according to the area from which the vulnerable person comes. The farther away the vulnerable patient is, the higher the cost of transportation and vice versa. A variation was applied to the food allowance at the very beginning of the care as well. But later on, the project team manager ordered systematic payment of allowance for all indigents who come for care.

≪ *Coverage includes transportation which varies according to distance, food, medical prescription and if it is serious, we give a bonus to refer the patient* ≫ IDI substitute Chief of health Center Tata1.

The data analysis of the HIF in Table 1 of the 38 facilities and nine (11) months of activity was carried out for the three types of variables (identified indigents, care, and transfer) according to the nature of the facilities. We found that the community health centers (CHC) identified 791 indigents, unlike the public health centers (PHC) and associative health centers (AHC), which identified only 945 and 251 indigents, respectively. As for the indigents taken in charge, it appears that the CHC and PHC have almost the same number of indigents taken in charge compared to the AHC which have a total of 120 indigents taken in charge. As for the number of indigents transferred, the CHC have a total of 25 indigents compared to 22 and 17, respectively, in the AHC and PHC. The visible difference between the number of indigents identified and those taken care of is explained by the fact that some of the vulnerable people identified were not treated because the system only provides for the care of the vulnerable when they fall ill.

**Table 1:** Health indigent fund.

**Variables**	**Private facilities**		**Public facilities**	**Total**
	**Associative health centers**	**Community health centers**	**Public health centers**	
Population served	383.554	42.210	156.651	582.415
Identified Indigents	251	791	945	1.987
Indigents taken in charge	120	439	446	1.005
Indigents transferred	22	25	17	64
Coverage (Indigents identified/population served)	0.07%	1.9%	0.60%	

Indigent coverage, calculated as the number of identified indigents concerning the population served by a given health facility, varies from 0.07 to 1.9% depending on the facility.

From the start of identification in May 2021 to December 2021, a total of 1,987 indigents were identified, of which 1,005 were managed and 64 referred to all 38 intervention health facilities under the IF. The remaining 952 did not use the IF. In relation to the population served in the intervention zones, the IF succeeded in identifying a small proportion of indigents (<1%) with a utilization rate of 67.6% of the total budget available for the care of the vulnerable at the time of this study.

It should be noted that there was no target related to the number of vulnerable people to be reached, as it was the passive and active identification processes that informed the care of the needy as they were identified.

The committee management fund agreed that the criteria for identifying the vulnerable were respected by all the actors involved in this process, namely the CHWs, health agents and community leaders and/or local councilors. At this level, all of them maintained that the members of the communities are suffering as a whole, but the most vulnerable ones, who are without any support other than that of goodwill, were identified by following the indications of the project.

≪ *It can be an elderly person, it can be a disabled person, a blind person, a hypertensive, a diabetic mother blind with many children… it is only vulnerable when he has no help, he has no generating activity that can help him pay the costs of his care, he has no child who can take care of him when he is sick*≫ IDI CHW CHERUBINS.

### Perceptions

Taking care of the vulnerable through the HIF is a highly humanitarian act in the eyes of the beneficiaries, leaders, health workers and other community members. The HIF has generated a great deal of enthusiasm within the communities, who have all appreciated this social and economic support. They encouraged the initiative and recognized that it is their (communities') responsibility that the project has taken on and therefore there is no reason to be reluctant about such an initiative.

≪ *It is a relief for everyone, it has facilitated access to care for many people who were suffering and others were dying like that without any medical assistance that things are sustainable*≫ IDI community leader Télimélé.

Many people are sick in the communities and they do not dare to go to the health facilities simply because they have no source of income and no alternative means to cover their medical expenses.

Some have had to sell their food reserves intended to feed all the members of the family to meet their medical needs. This recourse to the sale of the family's food reserve only occurs in case of ultimate necessity, as it is a technique that consists in cutting the food ration of all to meet medical expenses ≪ *for me, it was the family's expenses that I sold to send the seriously ill children to the health center* ≫, told us a widow we met in Korbè.

≪ *I often sell what we have here as a food reserve for the children's care, but if it's a big child or an adult, we boil leaves or buy a few tablets, because it's going to be expensive and I can't sell everything that is meant for family food to treat one individual*≫ IDI beneficiary of HIF Télimélé.

### Facilitators of HIF project implementation

The study showed that several factors reported by the various stakeholders facilitated the implementation of the project. Many, cited capacity building (having been trained in HIF management) of implementing actors, particularly CHWs and health workers (HWs). The training initiated for the CHWs contributed to the success of the sensitization sessions in the field and was considered a determining factor for some respondents.

≪…* it's the sensitization, we brought people together to do the sensitization and that is what facilitated the acceptance of the health indigent fund by the communities …* ≫ IDI chief center Dougayah.

The local recruitment of CHWs, the provision of means of transport for the agents' field activities coupled with the familiarity of these agents with the communities and their respective cultures were also cited as elements that facilitated the implementation of the project, ≪ *I really think that the CHW has a good knowledge of the localities and that helped a lot* ≫ said the head of the Hindè center.

In addition, there are also the supervisions that the project teams have carried out on each site and in collaboration with the field actors ≪…* The training is a very facilitating factor, the supervision and the recruitment criteria of the CHWs also facilitated us. These are the key criteria for the smooth running of this process of taking care of vulnerable people*≫. IDI health worker CARAD.

### Compliance of health agents, CHWs, and community leaders with the guidelines for the management of the vulnerable

All the participants in this study agreed that the criteria for identifying the vulnerable were respected by all the actors involved in this process, i.e., the CHWs, health agents and community leaders and/or local elected officials. At this level, all of them maintained that the members of the communities suffer as a whole, but the most vulnerable ones, who are without any support except for that of good will, were identified by following the indications of the project.

≪ *It can be an elderly person, it can be a disabled person, a blind person, a hypertensive, a diabetic blind mother with many children… he is vulnerable only when he has no help, he has no generating activity that can help him pay the costs of his care he has no child who can take care of him when he is sick*≫ IDI-CHW-CHERUBINS.

From the analysis of the results of this study, several points of view emerged in terms of compliance with the guidelines related to the care of the vulnerable. The first group, made up of health care providers and CHWs, maintained that the guidelines for the management of vulnerable persons, particularly the budget lines provided for by the project, were strictly respected, while community leaders and beneficiaries said that they did not know exactly what expenses were covered in the management of vulnerable persons. This state of affairs is due to the lack of sufficient information about the IF by these leaders or local elected officials. This lack of information could also be explained by a lack of involvement of all stakeholders (community leaders/local councilors) in the HIF management committee.

≪*… the transport depends on the distance and the food was fixed at 70.000fg per day and per patient…*≫ IDI Deputy health centre, Timbo.

There are some differences in the explanations given by the different actors involved in the implementation of the different activities in the care of the vulnerable. It is obvious that there are some differences on a number of aspects related to transportation and even the premium granted for eating between Conakry and the regions. In the case of the special region of Conakry, transportation is fixed at 50,000FG.

≪ *The transport is 50 000fg… the food is 85 000fg… we did not have reference cases, but in case of reference we give 500 000fg*≫ IDI CHW MSD Dixinn.

### Challenges encountered in the implementation of health indigent fund activities

At the start of the indigent care activities, some local councilors were reluctant to sign the care documents. This reluctance was since they were not well-informed about the validation process of these administrative documents. Still, some people remain very cautious when asked to sign an administrative document. Often, doubts of manipulation are feared. However, they may give in when explanations are given by trustworthy resource persons.

≪ *At the beginning, we had some difficulties with the Head of Sector…for the signature of the documents. He had refused to sign the documents; it was when Dr X came to intervene that he agreed to sign*≫ IDI CHW MSD Dixinn.

During the identification of the vulnerable, some reluctance among some indigent people was also relayed by participants. They refuted their vulnerable status and often claimed that they do not need someone to help them take care of themselves, as they considered this request for help to be an insult. Some perceived their economic situation as inevitable and therefore did not need anyone to assist them in this regard ≪ *eh others did not want to be identified as vulnerable, because they feel insulted or humiliated* ≫, according to a community leader met in Hindè. Similar cases were also encountered in Moriady, N'dita and Thiaguel Bori. But after some medical care for the first vulnerable patients, the echoes went away and those who were reluctant at the beginning came back to ask to be included in the list of the vulnerable.

≪ *All those who initially said they were not indigent ended up coming back when they realized that this care was serious* ≫ IDI local councillor N'dita.

Knowing the benefits of care, some vulnerable people decided to come to the center and declare that they are sick to benefit from the transportation and food bonuses provided for this purpose. These moments are embracing for the providers who are afraid of the attacks of these indigent people, and they often pretend to diagnose them and give them paracetamol tablets ≪ *When they understand that they will have the cost of transportation and food, others come to tell us that they are sick even if they are not. I have had cases like this and after the visit, I found that he was fine. I just gave him some paracetamols before I released him* ≫ IDI chief center Hindè.

The delay and the process considered complicated in the supply of funds by the health agents were mentioned by the health workers and the CHWs. The former criticizes this attitude and says that it could have an impact on the care of the vulnerable. Often, they take care of the indigent while waiting for project funds to arrive because vulnerable patients arrive without any money on them. They know that when they come, they will be taken care of. Often the delay in the supply of funds causes two major difficulties, including the breakdown of medicines in the facility because they have treated indigents with them and the reimbursement is delayed or sometimes the supporting documents have not been validated, there is no reimbursement in this case; this is a loss for the health worker and his facility. If they say that they are out of funds, the vulnerable and the community do not understand, for them, it is a refusal of the health agents, who want to make money with the health indigent fund.

≪ *There are no other difficulties except for the fact that they delay the health indigent fund, and we are obliged to treat the patients with the medicines we have; this creates shortages of medicines and affects our revenues… even that, you sometimes send t*≫ IDI chief center Korbè.

≪ *I sent reports, they [the project finance team] say that one financier is on leave, the other is on a trip, he is doing this or that…* ≫ Informal conversation chief center Djindjin.

Similarly, the delay in sending funds to the health workers and CHWs exposed them to communities who saw them as people who told nice stories about the HIF and could not keep their promises. They would then risk getting a bad reputation, as villagers might think they were keeping the money for themselves. As a result, some CHWs, in agreement with their chief center, have at times slowed down the identification of the vulnerable.

Such delays and complexity in obtaining and managing IFs lead to crises of trust between providers and community members. However, the project teams interviewed on the issue let it be understood that the delay in payment does not in any way translate into an obvious desire to prolong the process to the point of harming someone. It is just that for financial matters, rigor in the examination of the files is required. Throughout the discussions, they complained about the work of certain health agents, particularly in the production of financial reports, with a number of shortcomings.

The understanding of certain financial reports by the project team causes them a lot of problems. It is this scrutiny that takes up a lot of their time. Sometimes, they have to call back certain health agents to get more explanations before they can validate or reject a financial report.

≪ *Hum ah the case is complicated eh. We are not responsible for the delays very generally. It's the poorly prepared financial reports that cause all the delays. We have to deal with, sometimes call back the submitter of the report so that he can explain it better… Sometimes, we reject some because they are completely inconsistent and this even after telephone exchanges with them. We never reject a financial report without discussing it directly with the agent who submitted it*≫ IDI project implementer.

It is clear that in terms of establishment of first contacts and vulnerable cares such as their premiums allocation, were not as planned, because some were not reaching target people as recommended.

## Discussion

Poverty is the main barrier to accessing health services in many West African countries and certainly in Guinea. The emergence of COVID-19 in mid-December 2019 had been disruptive for social, health and economic sectors, especially in poor resourced settings like Guinea (4). The COVID-19 pandemic had reportedly affected healthcare utilization and communities' ability to pay for healthcare due to the lack of means due to the COVID-19 pandemic (as the confinement) (4) which led them to identify new but non-conventional healthcare-seeking behaviors. Participants reported a general decline in the use of health centers, chiefly in the primary curative consultation and vaccination services, due to a lack of trust between health workers and communities. The latter feared being deliberately contaminated by health workers. Thanks to this project, vulnerable people have access to free care.

The problem of health care access has been addressed in various ways. There are various attempts to remove financial barriers to accessing health care, from state-regulated free care to community-based health insurance schemes. There are also options such as tax revenue mobilization, user fee elimination, health insurance plans, waiver plans, demand-side financing such as vouchers and conditional cash transfers, purchasing, and outcome-based financing (OBF). All of these experiences occur in the absence of sufficient public spending on health care and are reported in numerous studies in several countries including Guinea (10–14).

Guinea's previous experience with the introduction of mutual health insurance is mixed. The general problem is that enrollment rates are modest. Research has shown that very low enrollment rates are not due to a lack of understanding of the schemes (15).

*The majority of participants gained a clear understanding of the concepts and principles behind Medicare. They appreciate the redistributive effects of the system… They point out the differences between traditional financial mechanisms and the principle of health insurance, the advantages and disadvantages of both*≫.

The low enrolment rates for previous insurance could be explained by community perceptions of assistance. Such assistance in some communities could be perceived as invective toward the populations directly benefiting. In addition, the lack of adequate awareness and the short duration of such projects, which are often pilot projects or funded by individuals such as NGOs, could be responsible for the low enrolment rate. In the project we studied, the choice was to introduce and implement a HIF, which is a variant of health equity funds (HEFs), where third-party organizations (often NGOs) are responsible for identifying the poorest and financing their access to care. These approaches have been well-documented in Cambodia and the experience has been reported in several publications (9, 16, 17). It is interesting to note, however, that HEFs have not “gone mainstream.” There are limited, mostly anecdotal, descriptions of similar pilots in Laos (18), in DRC (19, 20), Rwanda, Mali and Togo (8) and in Syria (21) s part of larger NGO-led health interventions, as well as in the DRC and Cameroon where they are part of NGO-led PBF schemes (6, 7).

This present project was at the same time an emergency and pilot project at the height of the COVID-19 pandemic to assist the vulnerable in their access to health care service. The project collaborated primarily with the Ministries of Health and Social Action, the National Health Security Agency (ANSS), and the Regional and District Health Directorates.

They offer an alternative to traditional waiver mechanisms by establishing a “third-party payer” organization that (1) identifies patients unable to cover their health care and (2) pays providers for services. In most cases, this body is independent of the health facility, and its funding sources are separate, giving it a degree of independence. The approach has spread rapidly in Cambodia, becoming a national policy in 2006. In French, the terms “*fonds d'indigents*” and “*fonds d'indigence*” (and more rarely the term “*fonds d'assistance*”) are most often used interchangeably to designate this type of financing system, whereas in Anglo-Saxon literature the term “health equity fund” is used almost systematically. However, a nuance is necessary: the term health indigent fund suggests that the target population is limited to indigent households and individuals, i.e., economically and socially excluded people. The term equity fund, on the other hand, is more inclusive and indicates that poor but not indigent households, who are unable (usually temporarily and partially) to pay for health care, can also benefit from the system. There are few documented experiences with equity funds in Africa. We found three similar initiatives, with third-party payment of care for those unable to pay. These are the medical assistance funds in Mali, the indigence funds in Mauritania and the hospital equity fund in Marovoay, Madagascar (22). We briefly detail them below. The Malian experience dates back to 2001, with the creation of the medical assistance fund in two reference centers in Sélingué (17, 23). The NGO Médecins sans Frontières accompanied the process and provided the program with a start-up fund. The medical assistance fund targeted two types of population: the assumption of responsibility for hospital care for the indigent and the granting of credit to people with temporary payment difficulties. In order to ensure sustainability, funding came from local resources: revenues generated by the sale of medicines, a contribution from the municipalities and a subsidy from the state. The results remained relatively weak, mainly due to a lack of resources and the lack of involvement of certain local actors. Indeed, after 3 years of existence, the Sélingué medical assistance fund had supported only 2% of the patients hospitalized. Among them, only 9% were considered indigent and fully exempt from their medical expenses.

This is the context against which we can discuss the HIF elements in this project. The installation of a functional HIF, as international experience shows, takes a long time and, eventually, government buy-in. It is far too early to say anything about government buy-in because HIF is still under development, one might say.

To build trust, which is essential for HIF to work, implementers and recipients need time. Mistakes will always be made, as we found that the committee of HIF (community leaders or local councilor) do not know the exact expenses and dedicated amounts that the HIF covers besides the medical fee exemption for the indigent. They say that often beneficiaries report that they received a sum of money in addition to care, but do not know why or how much.

The period covered can be considered a simple start-up exercise. Most implementers were more involved in identifying vulnerable people than in carefully managing the health indigent fund. There is a disconnect between the implementers in the communes and the managers of the funds: community leaders/elected officials have no say in the financial management of the fund.

However, the project has planned for these leaders to be sufficiently involved to ensure the best use of resources with the utmost transparency so that vulnerable people can fully benefit from all aspects of their care (medical, food and transportation costs). Their signature on project documents, which should function as a kind of guarantee of transparency, is then sufficiently reduced to a simple administrative task.

“*Beneficiaries often tell us when they return from care that they did not pay anything and that they got money, without saying how much or why”*

Some caregivers offer coffee to the needy who come for care instead of the dedicated premium “*he treats us for free and gives us coffee, I'm very happy…that's all he does for me every time, I leave*”.

These are issues that need attention, of course. But money is not the only issue—a better relationship between vulnerable people and health care staff is also a critical component of improving access to health care. Several informants noted that the dehumanizing attitude of some providers can scare off economically distressed patients. This demonstrates that financial access is only one dimension of access to health services and should be closely linked to improving the quality of services, which also involves respectful reception and treatment. Often it is the poorest who are identified as needy, but this does not mean that others are so rich that they can pay cash for medical services. Some resort to selling their equipment or animal goods to access care: “*If I get sick or my child gets sick, these are my roosters, I sell them to look for the products and sometimes it is not enough. Once, it was a pot that I had here, a big pot that I sold for the care of my child*.

The income situation in Guinea, and particularly in the rural areas where the project took place, does not make it useful to apply the term “catastrophic health event” (defined as out-of-pocket expenditures for health care that exceed 10% of annual household expenditures, 20% of an individual's income, or 40% of non-food expenditures) because it would be difficult to find non-catastrophic health events-it would provide a fictitious line of demarcation that does not help (24).

More importantly, as the first step on a long road to equal access to health care, there was a seemingly growing sense of community responsibility. Some people who come for care without money rely on the humanism of providers-but not always successfully: “*Some come for treatment on credit because they have no money; they plan to sell a rooster or a goat in order to return the doctor's money as soon as the purchase is made… But sometimes they do not succeed in making the sale in time, and the doctor starts chasing them around or asking for them, they will be ashamed of themselves, and soon they will no longer come until they have the cash,”* a CHW told us in Kélin.

On the beneficiary side, many found it appropriate to talk about other supports they did or did not receive, as long as they were getting the health care they sought. Thus, there can be no guarantee that the indigent always receives all the assistance to which they are entitled under HIF rules.

“*They do a lot for us; we don't pay anything for our medicines. Sometimes they also give us money;… I don't remember…”*

This seems very understandable given the depth of poverty in which some families live. Some HIF beneficiaries struggle daily for food and this heralds the dislocation of the family; each member wanders around all day in search of food; thus, perceived as mentally unstable in the eyes of the population. In such families, no one thinks of seeking care in case of illness. In terms of food, these people are usually supported by the goodwill of the community, which offers them food from time to time, “*we have nothing, it's thanks to this project that we think about going to the hospital for treatment, otherwise we don't even have food. Every child goes out in the morning for a walk, or looks for something to eat… some members of the community are even afraid of us; at home, here, everyone is sick, everyone is hungry…”*.

## Strengths and limitations

This study has a number of strong points that should be pointed out, which include the complex sampling procedure that took into account the variation in the selection of study participants, and the use of a hybrid approach (inductive and deductive) in the data coding process using the NVivo software.

It is also important to note a few limitations that should be highlighted, including the relatively low representation of health authorities and Memisa and FMG implementers among the participants interviewed. To minimize the impact of this, the participants from these different entities were selected according to their level of involvement in the project.

## Conclusion

Many participants agreed that poverty is the real barrier to the demand for health services. The project, with its indigent care component, marked a visible start in contributing to access to care for vulnerable people. The fear of the SARS-COV2 pandemic is gradually disappearing. In some places, the implementation of the HIF has created a certain awakening of the collective conscience and a spirit of solidarity toward some indigent people. The discussions about the HIF and the start of actual activities have brought about a news agency in the community. When the HIF covers medical expenses, community members join together to meet some of the needs of these vulnerable people, such as donating necessities. Some have gone so far as to build housing for a widowed woman and her children identified by the project as vulnerable.

≪ *In a locality here* [Thiaguel Bori]*, the communities, after identifying and taking care of a widow and her children, mobilized to build a two-bedroom house for them. Before the project, they lived in total oblivion [no one in the community was interested in their plight as destitute people]… They did the previous rainy season in this “small round hut with water running through it” in which the mother cooks and they slept there too…* ≫.

## Data availability statement

The raw data supporting the conclusions of this article will be made available by the authors, without undue reservation.

## Ethics statement

The research protocol has received approval L-080-CNERS-21 from the National Health Research Ethics Committee of Guinea. The patients/participants provided their written informed consent to participate in this study.

## Author contributions

The study protocol was developed by LB and MK and reviewed by WD, BC, and AD. The data were analyzed by LB. The first draft of the manuscript was written by LB and WD. The manuscript was critically reviewed by WD, JD, BC, AS, NA, and AD. All authors participated in the interpretation, read, and approved the final version of this manuscript.

## Funding

This project and study were funded by the European Union to the NGOs *Memisa* and *Fraternité Médical Guinée*, the Antwerp Institute of Tropical Medicine, the African Center of Excellence for the Prevention and Control of Communicable Diseases, and the Centre National de Formation et Recherche en Santé Rurale in Maférinyah, Guinea.

## Conflict of interest

The authors declare that the research was conducted in the absence of any commercial or financial relationships that could be construed as a potential conflict of interest.

## Publisher's note

All claims expressed in this article are solely those of the authors and do not necessarily represent those of their affiliated organizations, or those of the publisher, the editors and the reviewers. Any product that may be evaluated in this article, or claim that may be made by its manufacturer, is not guaranteed or endorsed by the publisher.
